# Public access defibrillation: Suppression of 16.7 Hz interference generated by the power supply of the railway systems

**DOI:** 10.1186/1475-925X-4-16

**Published:** 2005-03-15

**Authors:** Ivaylo I Christov, Georgi L Iliev

**Affiliations:** 1 Center of Biomedical Engineering, Bulgarian Academy of Sciences, Acad. G.Bonchev str., blok 105, 1113 Sofia, Bulgaria; 2 Technical University of Sofia, Department of Telecommunications, Kliment Ohridski str. 8, 1000 Sofia, Bulgaria

## Abstract

**Background:**

A specific problem using the public access defibrillators (PADs) arises at the railway stations. Some countries as Germany, Austria, Switzerland, Norway and Sweden are using AC railroad net power-supply system with rated 16.7 Hz frequency modulated from 15.69 Hz to 17.36 Hz. The power supply frequency contaminates the electrocardiogram (ECG). It is difficult to be suppressed or eliminated due to the fact that it considerably overlaps the frequency spectra of the ECG. The interference impedes the automated decision of the PADs whether a patient should be (or should not be) shocked.

The aim of this study is the suppression of the 16.7 Hz interference generated by the power supply of the railway systems.

**Methods:**

Software solution using adaptive filtering method was proposed for 16.7 Hz interference suppression. The optimal performance of the filter is achieved, embedding a reference channel in the PADs to record the interference. The method was tested with ECGs from AHA database.

**Results:**

The method was tested with patients of normal sinus rhythms, symptoms of tachycardia and ventricular fibrillation. Simulated interference with frequency modulation from 15.69 Hz to 17.36 Hz changing at a rate of 2% per second was added to the ECGs, and then processed by the suggested adaptive filtering. The method totally suppresses the noise with no visible distortions of the original signals.

**Conclusion:**

The proposed adaptive filter for noise suppression generated by the power supply of the railway systems has a simple structure requiring a low level of computational resources, but a good reference signal as well.

## Background

More than 35 years ago external cardiopulmonary resuscitation (CPR) and defibrillation were first described as effective treatments for sudden cardiac arrest. However, survival after out-of-hospital cardiac arrest is still poor. The American Heart Association (AHA) previously addressed this problem by emphasizing the importance of the 'chain of survival' [1]: early access, early CPR, early defibrillation, and early advanced life support. Because early defibrillation is the single most important intervention, the AHA challenged manufacturers to develop simple, low-cost automatic PADs for use at locations in which large numbers of people congregate: stadiums, airports,  railway stations, etc [2].

It has been reported that the chance of survival of a patient at a state of ventricular fibrillation decreases by approximately 10% with each minute that passes after the time of attack [3]. There also exists a probability of irreversible brain (cortical) damage due to systemic hypoxia after 5 minutes. However, response time for paramedics or emergency medical technicians to arrive on site with a defibrillator is often more than ten minutes, resulting in average survival rates of less than 5%. Widespread deployment of automated PADs is the only feasible method of achieving early defibrillation. The strategy for time reduction is that defibrillators can be used by non-healthcare personnel (for example, untrained bystanders and trained members of the staff at the public place) before the emergency medical services arrive.

A specific problem using the PADs arises at the railway stations. Some countries as Germany, Austria, Switzerland, Norway and Sweden are using AC railroad net power-supply system with rated frequency of 16.7 Hz [4]. According to the Official journal of the European communities[5] the interference is frequency modulated from 15.69 Hz to 17.36 Hz.

The power supply frequency is magnetically induced and can contaminate the electrocardiogram (ECG). It is difficult to be suppressed or eliminated due to the fact that it considerably overlaps the typical rhythms analysis spectra between 1 to 30 Hz of the ECG. The interference impedes the automated decision of the public access defibrillators whether a patient should be (or should not be) shocked, no matter of the high accuracy reported recently by some authors [[6,7] and [8]].

Schlimp et al [9] have tested two automated external defibrillators near high-voltage power lines run by the Austrian railway company with an AC of 16.7 Hz and 15 kV (overhead of railway tracks) or 16.7 Hz and 110 kV (overland). The reported errors are: i) failed operation due to artifacts; ii) misinterpretation of the artifact, resulting in failure to treat an underlying ventricular fibrillation and ventricular tachycardia or failure to recommend proper bystander action in the cases of underlying asystolia; iii) failure to identify clearly detectable rhythm and misinterpretation of the noise as motion artifact or ventricular fibrillation, resulting in a shock advice.

Kanz et al [10] have tested eleven automated external defibrillators with 16.7 Hz, 15 kV AC (S-Bahn) and a subway system powered with 750 V DC (U-Bahn). The authors have recorded 5280 tests concluding that high voltage AC interferes more (shock decision sensitivity reduction of 9.7% and no-shock decision specificity reduction of 10.6%) than low voltage DC (shock decision sensitivity reduction of 0.6% and no-shock decision specificity reduction of 1.4%). The interference is minimized, if patient position is parallel and electrode cables are perpendicular to overhead power lines. Four of the devices have demonstrated an unacceptable performance in respect of accuracy.

We could not find any material dealing with 16.7 Hz noise suppression generated by the power supply of the railway systems. That is why we referred to some standard real-time methods used for main interference (50 or 60 Hz) suppression, from the point of view whether it is possible to adopt them to the specific problem:

- The QRS suppression introduced by notch filters tuned to reject a band from 15.69 Hz to 17.36 Hz is so high that it makes them unacceptable.

- 'Comb' filters averaging in a time interval of the interference from 57 ms to 64 ms are over-smoothing the QRS complexes, making it sometimes undistinguished from the T wave.

- The good performance of the mains interference subtraction method [[11-13] and [14]] is mostly due to the correlation between the interference and the ECG sampling frequency. In the conditions of huge noise modulation the method seems inapplicable.

- Adaptive filtering described by some authors [[15,16] and [17]] does not disturb the electrocardiogram (ECG) frequency spectrum, but requires re-adaptation at any change of the normal ECG course, such as arrhythmias or appearance of an extra systolic ectopic beat. The re-adaptation period is characterized by the appearance of a tail of gradually attenuating unsuppressed interference. The same effect can be observed at any change of the interference frequency [16].

The aim of this study is to investigate the suppression of the 16.7 Hz interference generated by the power supply of the railway systems.

## Method

**Figure 1:**
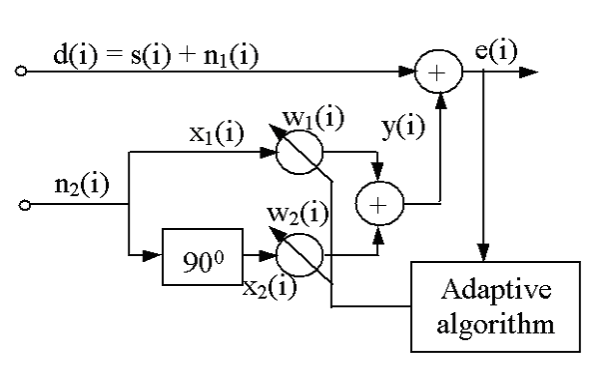
Block-diagram of adaptive filtering.

The problem of electric train noise suppression could be considered as a particular case of a more common problem of adaptive noise cancellation [18]. The adaptive filtering method is software based and could be added into the PAD computer algorithm, but does need a special hardware configuration (antenna) within the PAD to sample the electromagnetic interference signal without the ECG data for reference. We assume that a reference noise signal can be easily picked up and used. A block-diagram of adaptive filtering is shown in Fig. 1.

The primary input of the adaptive filter is the active channel ***d(i)***, which is a mixture of a clear ECG signal ***s(i) ***and the interference generated by electrical train ***n*_1_*(i)***, where ***i ***is the consecutive number of the sample:

***d(i) = s(i) + n*_1_*(i) ***    (1)

The noise has a sinusoidal waveform and it can be expressed as:

*n*_1_(i) = b*cos(2π*F/Fd*i + φ)     (2)

where ***b***, ***F***, ***Fd ***and ***φ ***are amplitude, frequency, sampling frequency and phase of the noise signal, respectively.

The reference input of the adaptive filter is a noise signal collected by an antenna:

*n*_2_(i) = a*cos(2π*F/Fd*i),     (3)

which differs from ***n*_1_*(i) ***only in amplitude and phase.

The task of the adaptive algorithm depicted in Fig. 1 is to adjust the coefficients ***w*_1_*(i) ***and ***w*_2_*(i) ***to compensate the amplitude and phase difference between ***n*_1_*(i) ***and ***n*_2_*(i)***. In other words, ***y(i) ***should resemble ***n*_1_*(i) ***as close as possible.

We rearrange the reference noise signal in the following manner:

*x*_1 _(i) = a*cos(2π*F/Fd*i)     (4)

*x*_2 _(i) = a*sin(2π*F/Fd*i)     (5)

where ***x*_2 _*(i) ***is the noise reference signal phase shifted on 90 degrees by Hilbert transform.

Conventionally the Hilbert transformer is realized as a FIR system [19]. The transition band reduction needs a very high order FIR system, which requires complicated computational structure. Considerable reduction in computational resources is achieved by all-pass structure which highest order is 5. In our structure the Hilbert transformer is realized as a parallel 3 all-pass sections. Two of them are intended to make the 90 degree shift and one - to prevent the distortion in the ***x*_1_*(n) ***signal. The order of the three sections is 4, 5 and 4 respectively. The advantage of this structure compared to the conventional one is that it allows very fine tuning of the signal phase and amplitude which is proved later with the experiments.

Then

*y(i) *= *w*_1_(i)*x_1_*(i) + w*_2_(i)*x_2_*(i)*,     (6)

and the error signal is formed as:

***e(i) = d(i) - y(i)*. **    (7)

We apply a Least Mean Square adaptive algorithm [20] with a cost-function ***e*^2^*(i) ***and finally get a system of two equations for adapting the coefficients ***w*_1_*(i) ***and ***w*_2_*(i)***:

*w*_1_*(i+1) = w*_1_(i) + 2*μ*e(i)*x_1_*(i) *    (8)

*w*_2_*(i+1) = w*_2_(i) + 2*μ*e(i)*x_2_*(i)*,     (9)

where ***μ ***controls the adaptation rate.

## Results

The adaptive filtering method was tested with ECG recordings taken from the AHA database. Each recording has duration of 30 min and includes two leads. The sampling frequency is 250 Hz and the resolution is 5 *μ*V*bit^-1^.

The experiments were performed by generating a sinusoidal-like noise ***n***_**1**_ having a main frequency of 16.7 Hz with frequency modulation of 20% for 10 s, and adding it to the ECG signals. The same noise but with different amplitude and phase from ***n***_**1**_was considered as a reference input ***n***_**2 **_for the adaptive filtering method.

**Figure 2:**
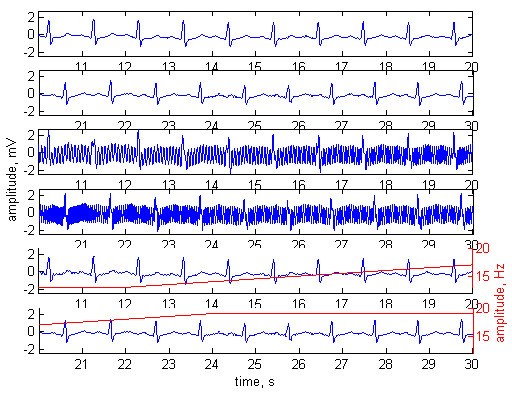
Adaptive filtering applied on normal sinus rhythm. Plots 1 and 2 – original ECG signal. Plots 3 and 4 – the ECG contaminated with ***n***_**1**_= 2 mV noise. Plots 5 and 6 – processed signal. The frequency modulation of the added interference is shown by red trace and red scale on the right. Interference amplitude at the reference channel ***n***_**2**_= 3 mV. Phase shift of 52° between ***n***_**1**_and ***n***_**2**_.

**Figure 3:**
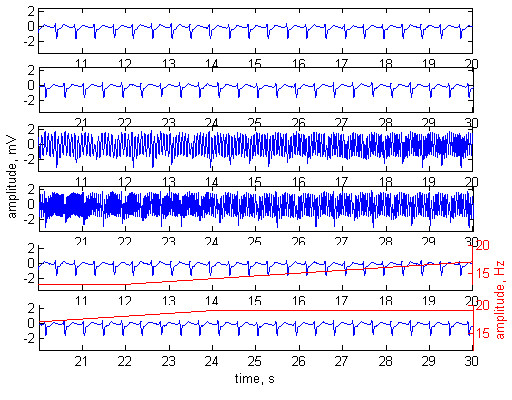
Adaptive filtering applied on a patient with symptom of tachycardia. Plots 1 and 2 – original ECG signal. Plots 3 and 4 – the ECG contaminated with ***n***_**1 **_= 3 mV noise. Plots 5 and 6 – processed signal. The frequency modulation of the added interference is shown by red trace and red scale on the right. Interference amplitude at the reference channel ***n***_**2 **_= 2 mV. Phase shift of 52° between ***n***_**1**_and ***n***_**2**_.

**Figure 4:**
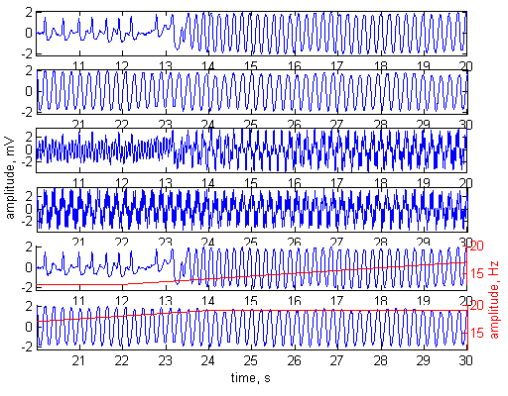
Adaptive filtering applied on a patient with ventricular fibrillation. Plots 1 and 2 – original ECG signal. Plots 3 and 4 – the ECG contaminated with ***n***_**1**_= 3 mV noise. Plots 5 and 6 – processed signal. The frequency modulation of the added interference is shown by red trace and red scale on the right. Interference amplitude at the reference channel ***n***_**2**_= 2 mV. Phase shift of 52° between ***n***_**1 **_and ***n***_**2**_.

The method was tested with normal ECG signals (Fig. 2), tachycardia (Fig. 3) and ventricular fibrillation (Fig. 4). The first two plots of any of the figures represent 20 s of the original ECG signal. Plots 3 and 4 present the ECG contaminated by a frequency-modulated noise. Plots 5 and 6 depict the processed signal as well as the frequency of the added interference, shown by red trace and red scale on the right.

The experiments were performed with phase shift of 52° between ***n***_**1**_ and ***n***_**2**_. The amplitudes of the noise were ***n***_**1**_= 2 mV, ***n***_**2**_= 3 mV in Fig 2, and ***n***_**1**_= 3 mV, ***n***_**2**_= 2 mV in Fig. 3 and Fig. 4.

**Figure 5:**
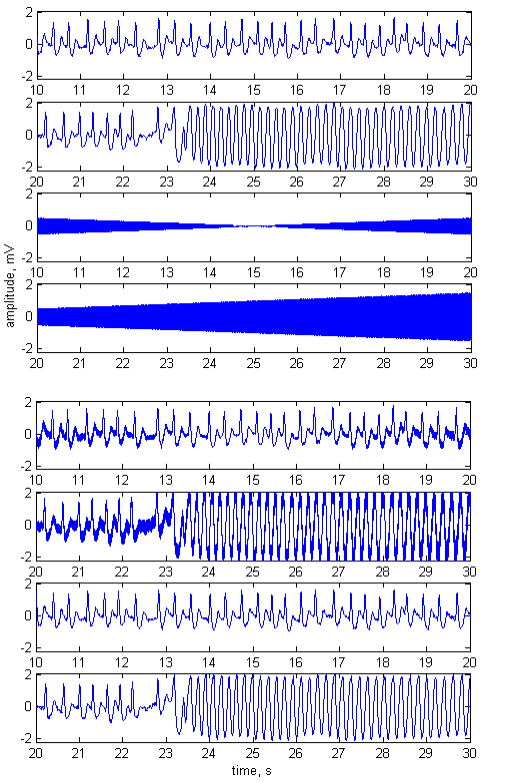
Plots 1 and 2 – original ECG with ventricular fibrillation. Plots 3 and 4 – sinusoidal noise with carrier frequency of 50 Hz (frequency modulated at a rate of 0.1 Hz*sec^-1^), and 3 mV amplitude (amplitude modulated at a rate of 0.2 mV*sec^-1^). Plots 5 and 6 -–the original ECG mixed with the 50 Hz noise. Plots 7 and 8 – processed ECG.

With no change of the coefficients of the Hilbert transformer, the same adaptive filter structure can be used for 50 Hz (or 60 Hz) power-line suppression. The first two plots in Fig 5 depict 20 s epoch of original ECG signal. Sinusoidal noise with carrier frequency of 50 Hz (frequency modulated at a rate of 0.1 Hz* sec^-1^), and 3 mV amplitude (amplitude modulated at a rate of 0.2 mV*sec^-1^), is presented in plots 3 and 4. The noise added to the original ECG is shown in plots 5 and 6. The same noise, but phase shifted with 90° is applied on the reference channel. The filtered signal is presented in plots 7 and 8.

## Discussion

Most often the interfering signal is presented not only with its carrier frequency but also with its third harmonic. The proposed adaptive filtering using a reference input can easily control signals with multiple harmonics by cascade or parallel structures based on the configuration shown in Fig. 1.

No visual distortions of the processed ECG signal due to the superimposed modulated interference are observed. The amplitude and phase difference between ***n***_**1 **_and ***n***_**2**_ affect mostly the initial adaptation time until the coefficients ***w*_1 _**and ***w*_2 _**reach some stable levels. With an adaptation rate of ***μ ***= 0.0001 the adaptation time is from 0.1 s to 20 s. The increase of ***μ ***cuts down the adaptation time but the filter results are getting worse.

After the initial adaptation time any amplitude and phase changes between **n**_**1**_ and **n**_**2**_ will be small and the system will succeed to readapt at a rate of ***μ ***= 0.0001. If a huge difference between **n**_**1**_and **n**_**2 **_appears, the re-adaptation period will depend on the gradient of the change, but in any case will be less than the initial adaptation time. The reaction of the system will be not to suppress enough the 16.7 Hz.

In an attempt to decrease the adaptation time we used a stepwise decrease of the adaptation rate after the start of the algorithm. First we make 30 iterations with ***μ ***= 0.1, then gradually reduce the step to 0.01 and 0.001 making 10 iteration at each, until a finally ***μ ***= 0.0001 is reached.

The starting number of 50 iterations was chosen empirically, considering that the amplitude of noise in one of the adaptive filter input should not be 3 times higher than the amplitude of the noise in the other input and the phase shift has to be lower than 60°.

The embedding of a reference channel in the PADs presents some practical difficulties. The antenna has to be designed in a way so that the reference noise does not significantly depend on the PADs position or possible people/object moving around the PAD during resuscitation. This needs to be subjected to further investigations following the publication. The final decision should be done after comparison to other methods, usually very complex and inflexible.

## Conclusion

The proposed adaptive filter for suppression of 16.7 Hz noise generated by railroad net power-supply system has an excellent performance and its simple structure requires a low level of computational resources. With no change of Hilbert transformer coefficients, the same filter structure can be used for suppression of the 50 Hz (or 60 Hz) power-line interference.
